# Highly reliable creation of floxed alleles by electroporating single-cell embryos

**DOI:** 10.1186/s12915-021-01223-w

**Published:** 2022-02-04

**Authors:** Monica F. Sentmanat, J. Michael White, Evguenia Kouranova, Xiaoxia Cui

**Affiliations:** 1 Genome Engineering & Stem Cell Center (GESC@MGI), Department of Genetics, Washington University in St. Louis School of Medicine, 660 S. Euclid Ave, St. Louis, MO 63110 USA; 2 Transgenic, Knockout and Microinjection Core, Department of Pathology & Immunology, Washington University in St. Louis School of Medicine, 660 S. Euclid Ave, St. Louis, MO 63110 USA

**Keywords:** Mouse models, Gene editing, Conditional knockout mice, CRISPR, Floxing, Functional genomics

## Abstract

**Background:**

Floxed (flanked by loxP) alleles are a crucial portion of conditional knockout mouse models. However, an efficient and reliable strategy to flox genomic regions of any desired size is still lacking.

**Results:**

Here, we demonstrate that the method combining electroporation of fertilized eggs with gRNA/Cas9 complexes and single-stranded oligodeoxynucleotides (ssODNs), assessing phasing of loxP insertions in founders using an in vitro Cre assay and an optional, highly specific and efficient second-round targeting ensures the generation of floxed F1 animals in roughly five months for a wide range of sequence lengths (448 bp to 160 kb reported here).

**Conclusions:**

Floxed alleles can be reliably obtained in a predictable timeline using the improved method of electroporation of two gRNA/Cas9 ribonucleoprotein particles (RNPs) and two ssODNs.

**Supplementary Information:**

The online version contains supplementary material available at 10.1186/s12915-021-01223-w.

## Background

Floxed alleles are an indispensable tool for the temporal and spatial regulation of gene expression in vivo and functional elucidation of sequence features/chromosomal regions identified in genomics studies. The availability of hundreds of tissue and cell-type specific Cre driver mouse lines and a wealth of historical data on floxed mice created via embryonic stem cell (ESC) technology have solidified the mouse as a key model organism [1–3]. With the development of programmable nucleases, single cell embryos are directly manipulated to create diverse mouse models, including floxed mice, with high germline transmission rates [4], circumventing the prerequisite for established ES cell lines.

Given that ssODNs are highly efficient donors for CRISPR (Clustered Regularly Interspaced Short Palindromic Repeats)-mediated small modifications [5], such as point mutations and short insertions of a loxP site or an epitope tag, the most straightforward method for floxing is to use two gRNAs and two ssODNs along with Cas9 protein, each gRNA/ssODN set delivering one loxP site. The two gRNA/ssODN sets are designed independent of, thus not limited by the distance between the insertion sites, although the efficiency of Cre-mediated recombination can be dependent on the size of the floxed region [6]. Yang et al. first reported highly successful floxing by microinjecting two ssODNs with in vitro transcripts (IVTs) of Cas9 and two gRNAs [7]. However, a large, multi-center study reported inconsistent results using the two gRNAs/two ssODNs approach [8], where only 11 out of 56 loci were successful, possibly due to some technical differences from Yang et al. [9]. In the meantime, at much smaller scales, multiple floxed models have been reported by microinjection of two gRNAs and two ssODNs along with Cas9 as IVT [10–12] or protein [13, 14] as well as electroporation of gRNA/Cas9 RNPs plus ssODNs of embryos [15], or oviducts [16], with the latter requiring a substantially higher concentration of RNPs and ssODNs. Yet, it is not clear how reliable the two gRNAs/two ssODNs approach is. Mouse work is costly, and it easily takes several months to confirm whether floxing is successful. There are two major competing events to floxing with two gRNAs: deletions between the two gRNA target sites and indels at each target site. An additional challenge is that in animals with both loxP sites, the two insertions can be *in trans* or even in different cells, in the case of a mosaic founder. An extra breeding cycle of about 10 weeks is often needed before one can be certain whether floxing is successful. Moreover, a repeat of a failed attempt can take another few months and double the cost without guaranteed success.

Alternatively, microinjection of long single-stranded DNA donors containing the target exon flanked by loxPs in combination with two RNPs has reported floxing efficiencies of 8.5–100% [17]. The technical challenge is to produce long single-stranded DNA (lssDNA) by synthesis beyond a few kilobases with high fidelity, when a large region needs to be floxed. lssDNA generated by transcription and reverse transcription is error prone due to the lack of a 3′ -> 5′ exonuclease activity necessary for proofreading [18]. Chemical synthesis and polymerase chain reaction (PCR)-based methods [19] are limited in length, and asymmetrical PCR (aPCR) is reported to generate lssDNA of over 15 kb [20] but still requires a large double-stranded DNA template to be available.

In short, a reliable method for floxing any desired length of sequences in a predictable timeline is still lacking. Here, we report the results on floxing 69 targets by electroporating single-cell embryos with two RNPs and two ssODNs, with successfully floxed regions ranging from 448 bp to 160 kb. We made two major improvements to maximize success rates. First, we used a highly effective in vitro Cre assay to assess phasing of the two loxP sites in founders so that confirmation of floxing can be obtained at the founder stage, and only animals with a floxed allele need to be bred to F1 generation [21]. Additionally, we developed a novel and reliable retargeting strategy to specifically insert a second loxP into an indel in phase with a loxP site in F0s, ensuring floxed F1 animals. The method combining the above strategies is highly dependable to generate multiple floxed F1 animals for a wide range of sequence lengths in approximately 5 months.

## Results

For each target, gRNAs were designed in introns flanking one or more exons that are critical for gene function or have coding sequences with lengths that are not the multiple of three. ssODNs were designed with a loxP site and a BamHI site inserted directly in the gRNA cleavage sites, flanked by 60 bases of homology sequences on each side. gRNA and ssODN sequences of targets are listed (Additional file 1: Table S1). gRNAs were one of the following formats: in vitro transcripts, chemically modified synthetic crRNA/tracrRNAs (CRISPR RNA/trans activating CRISPR RNA) or one-piece synthetic gRNAs, and ssODNs were with or without end protection by two phosphorothioate bonds at both 5′ and 3′ termini [22], as indicated. The ssODNs can be purchased at very reasonable prices from multiple vendors with a delivery time as short as 7–10 days. More importantly, the small size of ssODNs allows efficient electroporation along with RNPs into zygotes. More embryos can be manipulated in one session than by microinjection. The homology arms in ssODNs are around 60 bases each, allowing high throughput genotyping at each insertion site by next-generation sequencing (NGS) that is dependable and cost effective [22, 23]. RNPs and ssODNs were validated for efficient loxP insertion in Neuro-2a cells before embryo work, confirming activity and minimizing possible human errors by vendors or operators; see the “Methods” section. Validation data is also included in (see Additional file 1: Table S1). Among all different formats of gRNAs tested, the one-piece synthetic gRNAs with chemical modifications perform most efficiently and consistently. We observe a failure rate of approximately 5% across one-piece synthetic gRNAs tested, not limited to floxed models, from first round validation, and around a 2% failure rate after repeating, requiring a gRNA redesign. In our hands, end-protected ssODNs perform more efficiently and consistently in cultured cells, such as for validation. However, we do not have sufficient data in embryos for side-by-side comparison to ascertain whether modified ssODNs outperformed unmodified.

We collected data on the 69 floxing targets attempted in the past 4 years (Additional file 2: Fig. S1). Out of the 69 targets, 52 reached germline transmission with a floxed allele from at least one founder after one round of targeting, 40 of which are reported in Table 1 with permission to disclose from requesting principal investigators. All models were generated by electroporation of single-cell embryos. The total number of transferred embryos was from four sessions (each session uses up to 15 donor mice mated with studs and a minimum of four recipients at ~ 20 embryos/recipient) for each project. Potential founder animals were identified by the presence of loxP sequence via next generation sequencing (NGS) at both gRNA target sites. An example of NGS results is shown (see Additional file 3: Table S2). Detection of loxP site insertion and indel byproducts of non-homologous end-joining (NHEJ) at each target site is achieved in the same assay.

**Table 1:** Successful floxed projects

Target	Floxed distance (bp)	Embryos transferred	Live births	Pups with both loxPs	Floxed/bred
Abat	6614	492	62	6	1/3
Amdhd1	1935	598	138	2	1/2
Ar	3237	220	32	3	1/3
Atg5	4955	542	85	3	1/2
Atpif1	1207	327	104	7	1/2
Batf3	4151	435	70	2	1/1
Bptf	1126	214	24	2	1/2
Dcaf12	1273	619	142	1	1/1
Fcgr3	3225	426	91	7	2/5
Gfpt1	2619	430	44	2	1/2*
Gfpt2	1523	450	75	2	1/2*
Glut8	658	318	83	8	2/4
Gnpat	10737	660	92	4	1/4
Gpd2	825	422	124	2	1/2
H2-Dma	3760	613	160	7	3/7
Hal	1269	488	100	4	1/1
Ifi35	1273	467	91	11	2/3
Igh	988	483	86	5	2/3
Il4	6614	414	119	3	1/2*
Irgm1	2405	424	135	1	1/1
Itgax	1075	492	94	5	1/5
Kmt2c	1880	479	111	6	4/4
Ldlrad3	160942	534	125	4	1/3
Lrp1	1167	548	70	3	1/2
Lrpap1	448	315	89	3	1/2*
Mmp14	1822	447	66	4	2/4
Mxra8	5408	466	104	3	1/2
Nat10	1880	558	127	7	3/3
Nptn	1592	548	94	4	1/4
Per1	1920	430	81	3	1/2*
Sarm1	1218	483	101	3	1/2*
Slpi	1283	402	95	7	1/3*
Skida	3144	623	86	5	2/2*
Stmn2	1208	506	83	5	1/2*
Tcf19	2735	552	70	10	1/3*
Tifa	3002	581	117	5	1/2
Uba5	1177	438	52	3	1/3
Ube4a	9908	457	97	9	3/4
Ufsp2	632	449	45	2	1/2
Xylt1	7779	386	116	12	2/3

Floxed/bred refers to number of germline-confirmed floxed founders over the number of founders with both loxP sites that were bred to F1 generation

*Projects tested using in vitro Cre recombination assay. Positive Cre assay results correlated to successful germline transmission

**Fig. 1:**
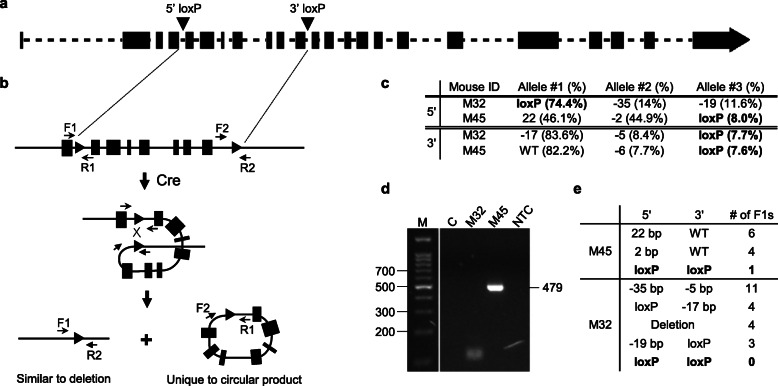
In vitro Cre assay predicts phasing of loxP insertions. **a** A target gene is floxed in introns 4 and 11 (arrowheads). **b** Schematic for in vitro Cre assay. The PCR amplicon by F2/R1 is unique to the circular product of Cre-mediated excision. **c** Two potential male founders, M32 and M45, are identified by NGS genotyping. **d** PCR products by F2/R1 resolved in an agarose gel. C: control, combined gDNA from animals positive for either 5′ or 3′ loxP site; NTC: no template control. **e** Genotyping of F1s from M32 and M45 confirms accuracy and sensitivity of the Cre assay

In order to predict whether a founder animal positive for both loxP sites carries a floxed allele before breeding to the F1 generation, we used the in vitro Cre assay [21], where purified genomic DNA was treated with recombinant Cre recombinase, followed by an excision-specific PCR amplified with primers F2 and R1, which face away from each other on the chromosome (Fig. 1A). In the circular excision product, primers F2 and R1 face toward each other and produce an amplicon containing one loxP sequence (Fig. 1B). Low genomic DNA concentration is used to promote intramolecular recombination. The excision-specific PCR is highly sensitive for the presence of circular product, and little background is detected in samples with loxP sites *in trans* (Fig. 1C and the “Methods” section). Cre assay-positive animals transmitted a floxed allele to F1 generation, even with relatively low percentage of loxP reads (Fig. 1D, E). Table 2 lists six out of seven projects at founder stage currently, all with at least one Cre assay-positive pup and highly likely to transmit a floxed allele. Among the projects in Tables 1 and 2, about 50% of the founders with both loxP sites carry a floxed allele, either confirmed by germline transmission or by positive Cre assay results. Together, 85% of the loci were successfully floxed by a single round of targeting.

**Table 2:** Floxed projects have at least one founder positive for the Cre assay, with germline transmission data pending

Target	Floxed distance (bp)	Embryos transferred	Live births	Pups with both loxPs	Positive/tested for in vitro Cre assay
Flt4	719	556	59	1 (1.7%)	1/1
Ccr7	2412	575	151	2 (1.3%)	2/2
Tmem135	726	555	166	11 (6.7%)	6/11
Dsg2	1649	265	38	3 (7.9%)	1/3
Prf1	2642	445	69	4 (5.7%)	4/4
Ifng	1547	483	70	3 (4.3%)	3/3

**Fig. 2:**
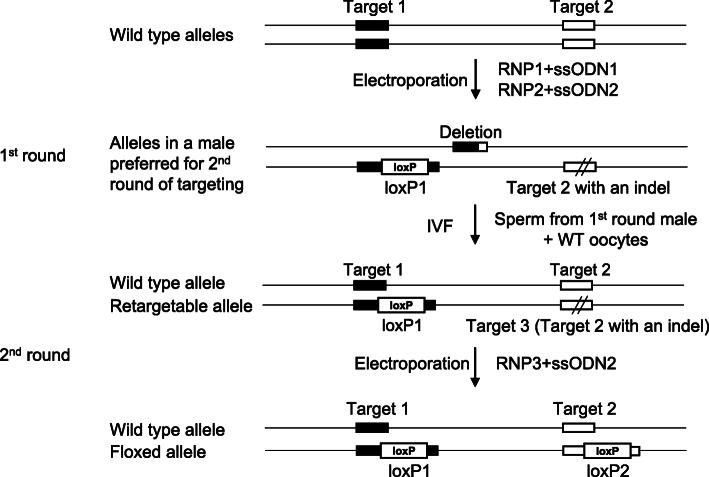
Schematics of 2-round targeting. A male with a deletion allele and an allele with loxP insertion in site 1 and an indel at site 2 (Target 3). Sperm from the male is used for IVF of wild type oocytes, and a new gRNA/Cas9 (RNP3) is electroporated with ssODN2 to insert the loxP specifically into Target 3. All resulting animals carrying both loxP sites have a floxed allele

Out of the 69 targets, ten loci failed to produce a floxed animal in the first round, four of which are listed (see Additional file 2: Table S3). Two projects did not generate livebirths with both loxP sites, and the other two each had one animal with two loxP sites that proven to be *in trans* judging by genotypes of F1 animals. A repeat of another four sessions will not guarantee a floxed founder. Instead, we took advantage of the presence of deletion alleles. All four projects had one or more F0 animals seemingly close to homozygous for one loxP insertion and having a small indel at the other insertion site with the protospacer adjacent motif (PAM) site intact, as shown in Table 3. The loxP site and the indel in each animal are highly likely in phase, and the other allele often contains a deletion between the two gRNA cleavage sites, which can be detected by a deletion PCR (not shown) but not by NGS at each individual site. A new gRNA, gRNA3, can be designed to specifically target the indel. Sperm can be collected from a male founder with such genotype and used for in vitro fertilization (IVF) of wild type oocytes. Electroporation of gRNA3/Cas9 RNP with the original ssODN for gRNA2 site for allele specific insertion efficiently results in a floxed allele in any live births with both loxPs (Fig. 2).

**Table 3:** Genotypes of F0 male candidates suitable for retargeting

Target	5′ end			3′ end		
	Total Reads	Allele #1	Allele #2	Total Reads	Allele #1	Allele #2
Nmnat2, F0	501	−2 (95.8%)	−3 (3.6%)	628	loxP (100.0%)	
Scn5a, F0^a^	1601	−27 (94.9%)	−15 (3.6%)	1975	WT (2.2%)	loxP (88.9%)
Slc38a9, F0	890	loxP (99.9%)		1624	−2 (92.2%)	
Zfhx4, F1	1210	WT (99.2%)		990	WT (56.7%)	loxP (42.5%)
Zfhx4, F0	193	loxP (97.4%)		154	−6 (99.4%)	−4 (0.6%)

In the first retargeting effort using a male F1 with wild type 5′ target site and a 3′ loxP insertion in the *Zfhx4* gene, two out of 40 live births from the second round targeting contained both loxP sites, among 22 positives for 3′ loxP and eight positives for 5′ loxP. One of the two with both loxP sites was positive for the Cre assay and transmitted a floxed allele (Table 4).

**Table 4:** Completed retargeting projects. All have transmitted the floxed allele. The NA designates “not applicable” to those targets where allele-specific gRNAs were used, and no Cre assay was performed

Target	Live births	Pups with both loxPs	Positive/tested for Cre assay	Germline transmission confirmed
Nmnat2	124	22	NA	Yes
Slc38a9	32	3	NA	Yes
Zfhx4	40	2	1/2	Yes

On the contrary, we obtained much more efficient retargeting by using a new gRNA against an indel. The new gRNA recognition sequences for retargeting two genes, *Nmnat2* and *Slc38a9*, are shown in Table 5. Fifty-eight out of the 124 pups from retargeting the 2 bp deletion at the 5′ target site of *Nmnat2* founder were positive for the 3′ loxP. The remaining 66 were 100% WT for both sites by NGS, most likely inheriting a deletion allele from the male founder that is undetected by NGS analysis of the target sites. Out of the 58 with 3′ loxP, 22 were also positive for the with 5′ loxP. None of the 124 animals had only the 5′ loxP site, confirming that the new gRNA did not recognize the wild type allele from the oocytes. In the 36 animals with only the 3′ loxP site, all but one had indels other than the 2 bp deletion at the 5′ target site, demonstrating high activity of the new gRNA targeting the indel. Germline transmission of a floxed allele was confirmed from these animals. For *Slc38a9*, three out of 32 live births had both loxP sites and germline transmission of the floxed allele was also confirmed. Similarly, if we retargeted the *Zfhx4* founder (*Zfhx4* F0) in Table 3 at the 5′ site for the 6 bp deletion, we would likely have obtained more floxed F1 animals, and the canceled Scn5a project can also be similarly retargeted.

**Table 5:** gRNA3 sequences used for retargeting. NGG, the PAM site following 20 bp of spacer sequence for each gRNA. Red dashes, --, represent deleted bases from the wild type spacer sequences and upstream bases added at the 5′ end of the spacer sequences are in red. Zfhx4 was retargeted using the original gRNA against the wild type target. All gRNAs were ordered from IDT as one-piece synthetic gRNAs

Target	Initial gRNA	gRNA targeting indel
Nmnat2	5′-TCAAGCAACGGTAATGCTGC**NGG**	5′-TCTCAAGCAACGGTAATGC--C**NGG**
Slc38a9	5′-GTCCTGTGCGGCCTTGTATT**NGG**	5′-TTGTCCTGTGCGGCCTTG--TT**NGG**
Zfhx4	5′-TAATTAGGCCGACATGAACG**NGG**	5′-TAATTAGGCCGACATGAACG**NGG**

In conclusion, targeting an indel in phase with a loxP site, when available, is a more efficient retargeting strategy. However, in projects without a male founder carrying a loxP insertion and an indel *in cis*, retargeting a wild type site, such as in *Zfhx4,* is still feasible but results in fewer floxed F1 animals.

## Discussion

In this study, we used electroporation to deliver two CRISPR RNPs and two ssODNs into single cell embryos for creating floxed alleles with a success rate of 85% (59/69) from a single round of four mouse sessions and 100% (63/63), if supplemented with a second round targeting (Additional file 2: Fig. S1). Floxed alleles require the insertion of two loxP sites into the same copy of the target gene after two double strand breaks are introduced. Various editing events can happen and compete with the formation of a floxed allele. Mainly, indels mediated by nonhomologous end joining can occur at each target site, and deletions can form between the two cut sites. An inverse relationship has been reported between deletion frequency at two targets and their distance from each other, where gRNA targets distanced < 10 kb from each other can result in up to 20–30% deletion products spanning both loci [24]. An allele can also have an indel at one site and loxP inserted in the other. The different editing outcomes are listed (see Additional file 2: Fig. S2). So floxing is a relatively low efficiency editing event and can be difficult to obtain under nonoptimal conditions. Here, we report a reliable floxing strategy routinely used by our center with success: electroporating embryos with validated reagents, assessing loxP phasing using an in vitro Cre assay, and, when needed, retargeting a male founder with a single loxP site via IVF.

Prior to embryo electroporation, validating synthetic gRNAs and ssODNs by transfecting Neuro-2a cells and analyzing the target sites using NGS is an important step to avoid unnecessary waste of time and money by faulty reagents or human errors. Even though the majority of reagents pass validation, the simple protocol is a worthy effort, given it takes at least 6 weeks to find out about a failed mouse session.

Among the 69 targets, we obtained various numbers of animals with both loxP sites, up to 12% of live births, from single round targeting. We observed about 50% of founders with both loxP sites carried a floxed allele, so when there is only one or two potential founders, floxing may not be successful. Confirmation by germline transmission takes 8–10 weeks from the time founders are genotyped. Instead, we used the in vitro Cre assay, where recombinant Cre is incubated with low concentrations of genomic DNA to encourage intramolecular reaction, so that Cre-mediated excision only occurs between the loxP sites *in cis*, forming a deletion product and a circular product. The circular product is detected by an excision-specific PCR with high sensitivity, even in founders with relatively low percentages of loxP-containing reads (Fig. 1). All animals positive in the in vitro Cre assay transmitted a floxed allele, when bred. On one hand, only founders positive for the Cre assay need to be bred to the F1 generation. More importantly, if no animal has a floxed allele, the second round targeting can be planned right away, saving up to 10 weeks of time. Whereas the in vitro Cre assay is sensitive enough to detect even a low percentage floxed allele in a founder, about 5% of targets turned out to be difficult to detect, including a very large floxed region (> 400 kb, greater than the average size of gDNA fragments, a target not reported here) and targets with multiple high homology sequences elsewhere in the genome. A negative Cre assay on all founders with both loxP sites is not conclusive, and multiple founders should be bred for confirmation if available, given about 50% of animals with both loxP sites carry a floxed allele.

Among the 69 targets we tried to flox, 10 did not result in a floxed allele with the first four electroporation sessions. Recognizing that many alleles with a single loxP insertion have an indel *in cis*, we used a third gRNA to target the indel specifically in embryos obtained by IVF with sperm from a male founder and wild type oocytes. Half of the fertilized eggs resulting from IVF have one wild type allele and an allele with a loxP at one target site and an indel at the other. Specific insertion of loxP into only the indel ensures that F1 animals with both loxP sites carry a floxed allele (Fig. 2). A higher percentage of floxed F1 animals were obtained when retargeting was against an indel in phase with a loxP at the second site (22/124, 3/32) than against the wild type sequence (1/40). The latter resembles a sequential targeting strategy, where one set of RNP/ssODN is electroporated to obtain male founders with one loxP insertion and sperm will be used in IVF for single cell embryos, which will then be electroporated with the second set of RNP/ssODN. The sequential strategy circumvents the competition from deletion alleles but is obligated to have a second round of targeting. Essentially, two-round targeting breaks down the relatively low efficiency floxing event into two relatively high efficiency events: insertion of a single loxP mediated by a CRISPR RNP and an ssODN. To date, all four attempted retargetings were successful.

Even though all four projects reported here had clearcut retargetable alleles in males, there are many more male founders with one loxP site and one or more indels at the second site at frequencies < 90% for each indel. In these cases, it can be difficult to decide which male founder to retarget. Additionally, larger indels at gRNA cleavage sites have been reported [25], which may not be detectable by NGS and can mislead the interpretation of genotyping. One solution is to breed the founder to the F1 generation and retarget F1 males with a loxP site and an indel. However, an additional 3 months would be added to the timeline. Alternatively, sperm can be frozen from all males with a loxP site and one or more indels at the second site. A straw of sperm from each male could be used for in vitro fertilization of wild type oocytes to obtain a small number of blastocysts to genotype and determine phasing of the loxP site and the indel. This way, retargeting will only be done with sperm of confirmed F0s, that have the desired genotype in phase without adding to the timeline. This genotype confirmation step also ensures sperm samples were collected from the correct animals.

A usable PAM site is needed for retargeting at an indel. Most of CRISPR-mediated indels are small. The original PAM site was maintained in all retargeted projects reported here, primarily because we observe small indels most commonly by CRISPR editing. However, it is possible that a deletion removes the PAM site, and there is no convenient one nearby. Testing individual blastocysts from multiple founder sperm samples via IVF increases the chance of identifying a retargetable animal with a PAM site. Yet, it would be prudent at the design stage to pick out sites with nearby PAMs when possible in the event a larger indel compromises retargeting potential. The original ssODN can usually be used for retargeting, unless the indel significantly reduced one or both of the homology arms in the ssODN.

For decades microinjection has been used to create transgenenic animals, and then for nuclease-mediated embryo manipulation [26]. It remains the go-to method for delivery of large molecules, such as DNA plasmid, long single-stranded DNA, and mRNAs. However, it is usually the bottleneck for throughput, taking microinjectionist hours under the scope to inject a few hundred embryos. For protein molecules, small DNA or RNA molecules, electroporation of single cell embryos has been very effective using various apparatuses [27–30]. Electroporation is much less labor intensive and time consuming than microinjection, and the conditions are more reproducible from operator to operator. The limit of the number of embryos to be electroporated in a given day is determined by the availability of embryos and recipients rather than available time under the scope and skilled microinjectionists. Higher embryo survival and thus birth rates are consistently observed after electroporation compared to microinjection owed in large part to less physical damage to the embryo [28, 29, 31]. If combined with using HyperOVA [32] and IVF to produce fertilized eggs, it is possible to electroporate large numbers of eggs with RNPs plus ssODNs in one day to obtain over 100 live births. Additionally, in our hands and those of others, electroporation of RNPs consistently results in higher editing efficiency [28, 33]. The electroporation protocol transfer to a second mouse core on campus was straightforward and produced similar success in floxing using validated reagents, NGS genotyping and in vitro Cre assay for seven targets (not shown). Five of the targets reached germline transmission with one round, one with Cre assay-positive founders and one being retargeted.

**Fig. 3:**
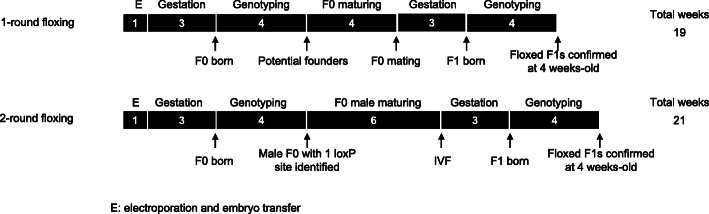
Timeline comparison for one or two-round targeting strategies

The timeline to identify F1s with a floxed allele is around five months with either one-round or two-round targeting (Fig. 3). Starting from electroporation, it takes about 8 weeks to identify floxed founders by using the in vitro Cre assay for one-step floxing and 20 weeks to reach F1 generation with confirmed germline transmission or to validate multiple floxed animals resulted from a second-round of targeting.

A common alternative method is to use long single-stranded DNA (lssDNA) donors [17], which have been reported to be efficient to flox a relatively small region. lssDNAs can be difficult to synthesize, require a double-stranded template, and are limited by size. There are times floxing more than a few kb is necessary, such as genes with multiple splicing isoforms needing more than one exon to be floxed. When using two gRNAs and lssDNA to flox a gene, deletion and individual indels still occur as competing events. One attractive solution to overcome these competing events is to use a nuclease-dead Cas9 to knock-in the loxP sites, such as prime editors [34], once targeting efficiencies for the system improve.

The two RNPs/two ssODNs method, delivered in one round or two, is highly flexible with the size of the region to be floxed. The largest region floxed in this study was 160 kb. If necessary, much larger sequences can in theory be floxed using the method, given the two loxP insertions are generally independent. Synthetic gRNAs and ssODNs can be obtained commercially and validated in cultured cells within a month at relatively low cost. The small size of ssODNs is compatible with efficient embryo electroporation and high-throughput NGS genotyping. Combined with the in vitro Cre assay and optional second round targeting, by electroporating two CRIPSR RNPs and two ssODNs, one can reliably obtain several floxed F1 animals in roughly five months either by one round or two round targeting.

## Conclusions

The method combining embryo electroporation with validated RNPs/ssODNs, assessing phasing of loxP sites in founders and optional retargeting via IVF ensures cost-effective creation of floxed F1 animals of wide range of sequence lengths in a predictable timeline.

## Methods

gRNAs were designed using an in-house algorithm that incorporates specificity scores from Zhang lab [35] and activity prediction scores by Doench lab [36] as well as single nucleotide polymorphism (SNP) search and primer design. The specificity score is the primary criterion for selection of a gRNA design, selecting those with specificity scores > 85. When necessary, one can choose a different region to design gRNAs when specificity score is too low at a given site. We have not observed high correlation between Doench score and synthetic gRNA activity and only avoided gRNA design with extremely low Doench score, such as 0.2 and below, when other designs with comparable specificity are available. Mouse SNPs within the 23 bp genomic targeting region of the gRNA were queried using dbSNP and, those with SNPs, cross-referenced with the Mouse Genomes Project dataset to query if a SNP has been reported in a non C57BL/6 target strain [37]. Synthetic gRNAs were purchased as synthetic RNAs from IDT (Coralville, Iowa) or Synthego (Menlo Park, CA).

IVT templates of gRNAs were generated by PCR combining two overlapping oligos, one containing T7 promoter and 20 nucleotides of spacer sequence (5′- aaaaTAATACGACTCACTATAGGnnnnnnnnnnnnnnnGTTTTAGAGCTA) and the other containing sgRNA backbone (5′-AAAAAAAGCACCGACTCGGTGCCACTTTTTCAAGTTGATAACGGACTAGCCTTATTTTAACTTGCTATTTCTAGCTCTAAAAC), together with a T7 forward (5′-AAAATAATACGACTCACTATAGG) and a backbone reverse (5′-AAAAAAGCACCGACTCGGTGCCA) primer. PCR was performed using AccuPrime HiFi Taq polymerase (Invitrogen) under the following conditions: 95 °C, 2 min, and then 35 cycles of 95 °C, 30 s; 60 °C, 30 s; and 68 °C, 40 s. PCR product was purified by QiaQuick PCR purification kit (Qiagen).

In vitro transcription reactions using the above purified PCR products as templates were done using HiScribe T7 Quick High Yield RNA Synthesis Kit (NEB). The sgRNAs were purified with SPRI beads (RNAcleanXP, Beckman Coulter) and quantified by using Qubit BR RNA assay for Qubit (Thermo Fisher).

Cas9 protein was purchased from QB3 MacroLab at UC Berkeley.

ssODNs contain a loxP site and usually also a restriction site, such as for BamHI, inserted directly at the cut site and 60 nucleotides of flanking homology arms with two phosphorothioate bonds at each end of the molecules. The ssODNs were ordered through IDT as desalted ultramers. The two ssODNs for a given floxed project should be designed on the same strand to minimize annealing between the two ssODNs at loxP sites. All retargeted animals (F0 and F1s) used the originally designed ssODNs listed in Additional file 1: Table S1.

Validation in cultured cells using 100 μM gRNAs were mixed with 40 μM Cas9 protein to form ribonucleoprotein (RNP) particles and combined with 50 μM of ssODN. Each RNP/ssODN set was then nucleofected alone or combined into mouse Neuro-2a cells, using SF solution and DS137 program on a 4D Nucleofector X unit (Lonza, Basel, Switzerland). After 24–72 h, cells were collected for NGS analysis (see below).

### Mouse husbandry

All animals at Washington University in St. Louis are housed under SPF barrier conditions in AALAC-accredited facilities. The Pathology Transgenic Core mice are housed in an enhanced barrier facility known as the RSI (Resource for the Study of Immunity). These animals are free from MNV, in addition to meeting standard requirements of Wash U SPF facilities. All required breeding, experiments and interventions are included in IACUC approved protocols. The Department of Comparative Medicine provides basic husbandry in accordance with their SOPs.

### Electroporation conditions

Each 10 μl of electroporation sample contains 12 μg of Cas9 protein, 2 μg of each gRNA, and 100 pmol (~ 5 μg) of each ssODN. Thirty to forty prepared single-cell embryos in 10 μl of OPTI-MEM are mixed with 10 μl of sample before being loaded into a 1-mm electroporation cuvette. With a Biorad Gene Pulser Xcell electroporator, we use 2–6 pulses of 30 V for 3 ms with 100 ms internals, as described by Chen and colleagues [28].

### Embryo manipulation

Fertilized single-cell embryos obtained from mating male and female mice from C57BL/6N or C57BL/6J were obtained through natural mating at 0.5 dpc, treated with Hyaluronidase and Tyrode’s solution to weaken the zona. Each session involves up to 15 donor mice with a minimum of 4 recipients. Post electroporation, the embryos are allowed to recover in KSOM in a CO2 incubator at 37 °C for a few hours before transfer to the oviducts of pseudo pregnant recipients. Embryo transfer into the oviduct is unilateral, at ~ 20 embryos/recipient.

### IVF for retargeting embryos

Sperm was isolated and capacitated in MBCD medium, one caudal epididymis in 250 μl medium. Oocytes were isolated from hormone primed females of C57BL/6N or C57BL/6J (PMS/HCG) and placed into 90 μl drops of GSH medium; 5 μl of sperm was added to the oocytes in GSH medium and cocultured for 5–6 h. Oocytes/embryos were then washed through 3 drops of Cooks KRVF medium and placed on a depression slide with M2 for visual assessment of fertilization. Embryos were electroporated with CRISPR reagents and cultured overnight. The next day, two-cell embryos were transferred into d0.5 pseudopregnant females, 15–20 per mouse unilaterally.

### NGS-based genotyping

Transfected Neuro-2a cells as well as tail clips of pups born to electroporated embryos were lysed in QuickExtract Solution from Lucigen (Madison, WI), following manufacturer’s instructions. The target region is PCR amplified by tailed primers appended with 5′-CACTCTTTCCCTACACGACGCTCTTCCGATCT-3′ for forward and 5′-GTGACTGGAGTTCAGACGTGTGCTCTTCCGATCT-3′ for reverse to genomic-specific primer sequences (Step1 PCR, see Additional file 1: Table S1), which allows unique indexes and Illumina P5/P7 adapter sequences to be added in a second round PCR. PCR amplifications were performed with EconoTaq PLUS GREEN 2X Master Mix or MyTaq Red Mix (Bioline), according to the manufacturer protocol. Indexing of the Step1 PCR product was performed by using 0.1X volume from Step1 with indexing primers (0.1 μM final concentration for each) and melting at 94 °C for 2 min, followed by five cycles of 94 °C for 30 s, 54 °C for 30 s, and 72 °C for 40 s. We generated 2 × 250 reads with the Illumina MiSeq platform at the Center for Genome Sciences and Systems Biology (Washington University). The extracted FASTQ files are analyzed by using a Python-based alignment script which outputs read counts of top alleles as illustrated in Table S2 [38]. Details can be found in our previous study [39].

### In vitro Cre-mediated recombination

Genomic DNA is prepared from tail snips using the DNeasy Blood and Tissue Kit (Qiagen). In a final volume of 20 μl, combine 2 μl of 10x Cre buffer, 0.1 μg of genomic DNA, and 10 units of recombinant Cre recombinase (NEB). Incubate at 37 °C for 30 min. The use 1 μl of Cre reaction as PCR template, amplify using F2 and R1 primers (Fig. 1B). Sequence the PCR products to confirm.

### In vitro validation of the new gRNA for retargeting an indel in phase of loxP insertion

PCR product of the indel-containing target site is amplified from genomic DNA of the founder to be retargeted, purified, and incubated with the new RNP. The digested reaction is resolved on the gel.

## Supplementary Information

**Additional file 1: Supplemental Table 1.** (Additional file 1.xlsx) Table S1. gRNA and ssODN sequences and validation in N2A cells.

**Additional file 2: Supplemental methods, Supplemental Figs. 1 & 2** and Table 3. (Additional file 2.docx) **Fig. S1.** Outcome of the 69 targets included in this study. Fig. S2 Different possible editing outcomes after two RNPs and two ssODNs are introduced into the embryos. Table S3: Four projects that failed to produce a floxed founder in the first round.

**Additional file 3: Supplemental Table 2.** (Additional file 3.xlsx) Table S2: Examples of NGS genotyping for founder identification. WT and loxP represent the wild type target site and exact 34 bp of loxP site sequences. loxP-containing alleles are boxed. Positive numbers under each allele represent number of base pairs of an insertion, and negative numbers, base pairs of a deletion. Animals with no reads at both sites most likely have biallelic deletion between the two target sites, and no reads at one site implies larger indels than NGS can reveal. Animals with a single loxP insertion are those with underlined animal IDs, and IDs of animals with both loxP sites are in bold. Animals with both loxP sites are potential founders and are candidates for in vitro Cre assay. Genotypes likely caused by NGS error are in grey.

## Data Availability

Not applicable.

## Abbreviations

Floxed: Flanked by loxP
ssODN: Single-stranded oligodeoxyribonucleotide
RNP: Ribonucleoprotein particle
ESC: Embryonic stem cell
CRISPR: Clustered regularly interspaced short palindromic repeats
IVT: In vitro transcription/in vitro transcript
lssDNA: Long single stranded DNA
PCR: Polymerase chain reaction
crRNA: CRIPSR RNA
tracrRNA: Trans activating CRISPR RNA
NGS: Next-generation sequencing
NHEJ: Nonhomologous end joining
PAM: Protospacer adjacent motif
IVF: In vitro fertilization
SNP: Single nucleotide polymorphism
