# Supplementary material for: Highly reliable creation of floxed alleles by electroporating single-cell embryos
Source: BMC Biol. 2022 Feb 4;20:31. doi: 10.1186/s12915-021-01223-w (PMC8815186; doi:10.1186/s12915-021-01223-w)
Supplement: Supplementary file 2 — Additional file 2: Supplemental methods, Supplemental Figs. 1 & 2 and Table 3. (Additional file 2.docx) Fig. S1. Outcome of the 69 targets included in this study. Fig. S2 Different possible editing outcomes after two RNPs and two ssODNs are introduced into the embryos. Table S3: Four projects that failed to produce a floxed founder in the first round. [file 12915_2021_1223_MOESM2_ESM.docx]

Additional file 2

**Highly reliable creation of floxed alleles by electroporating single cell embryos**

Monica F. Sentmanat^1#^, Michael J. White^2#^, Evguenia Kouranova^1^, Xiaoxia Cui^1*^

^1^Genome Engineering & iPSC Center (GEiC), Department of Genetics, ^2^ Transgenic, Knockout and Microinjection Core, Department of Pathology & Immunology, Washington University in St. Louis School of Medicine, 660 S. Euclid Ave., St. Louis, MO 63110

# Co-first authors

* Corresponding author: [x.cui@wustl.edu](mailto:x.cui@wustl.edu)

Supplemental Methods

Validation of reagents

Creating a floxed allele requires insertions of two loxP sequences on the same chromosome. The efficiency of floxing is naturally lower than introducing a point mutation or a single loxP insertion because of competing editing events such as deletions between the target sites and indels at each target site. We recommend electroporating 400-500 embryos with two sets of validated RNPs/ssODNs and aim at 100 live births per project. Given the high cost and long cycles of mouse work, it is important to make sure sgRNAs and ssODNs are functional and to also minimize human errors related to reagent production, ordering or labeling. Most, but not all, synthetic gRNAs are active. We also experienced vendor errors, such as a batch of gRNAs from one vendor repeatedly failed validation in transfected cells, and gRNAs with the same designs from a different vendor all worked in the same validation assay. Validation in a mouse cell line is simple and quick (see Methods). Data can be obtained within days. More importantly, it is a small cost to help prevent wasting multiple mouse sessions and weeks of lost time. **Table S1** shows the validation data for reported targets. We use an arbitrary 15% NHEJ for gRNAs and 2% of loxP insertion as cutoff, although most sgRNA/ssODN combinations perform significantly better. IVTs and two-piece gRNAs had more variable activity levels in N2A cells than single-piece synthetic gRNAs.

Choice of founder animals for retargeting

When retargeting is needed, a male founder carrying a deletion allele and an allele with an in-phase loxP site at one end and a retargetable indel at the other (NGS showing close to 100% loxP at one end and similar percentage of a given indel at the other, as the example in **Table S2**) is preferred. Sperm of the founder will be collected at 10 weeks of age and used for IVF of wild type oocytes for heterozygous single-cell embryos for retargeting at the indel. Fifty percent of the embryos will carry one allele of the indel. Only the allele with the indel will be targetable. All animals born with two loxP sites carry a floxed allele. If such a male founder is not available, a male founder with a deletion allele, a loxP at one site and wild type at the second site can be used for IVF. Not all animals born to retargeting of a wild type second site with both loxP insertions have a floxed allele in this case, that is, the two loxP sites can still be *in trans*. To avoid having to use F1 males for retargeting and adding about 10 weeks to the timeline, sperm can be frozen down from all male founders with a loxP site and indels at the second site. A straw of sperm from each founder can be used to in vitro fertilize wild-type oocytes, and the resulting fertilized eggs can be cultured in vitro to morula or blastocysts, which can be genotyped individually to identify the founders carrying an allele with a loxP site and an indel *in cis*. This way, sperm samples are genotype-confirmed before retargeting, and founder males without a deletion allele can also be identified with confidence to be suitable sperm donors for retargeting. In the rare cases that no males apparently homozygous for one loxP insertion or only females have a loxP insertion, breeding a loxP-positive animal to obtain F1 males for IVF is necessary for second-round targeting.

**Table S3** Four projects that failed to produce a floxed founder in the first round. ****


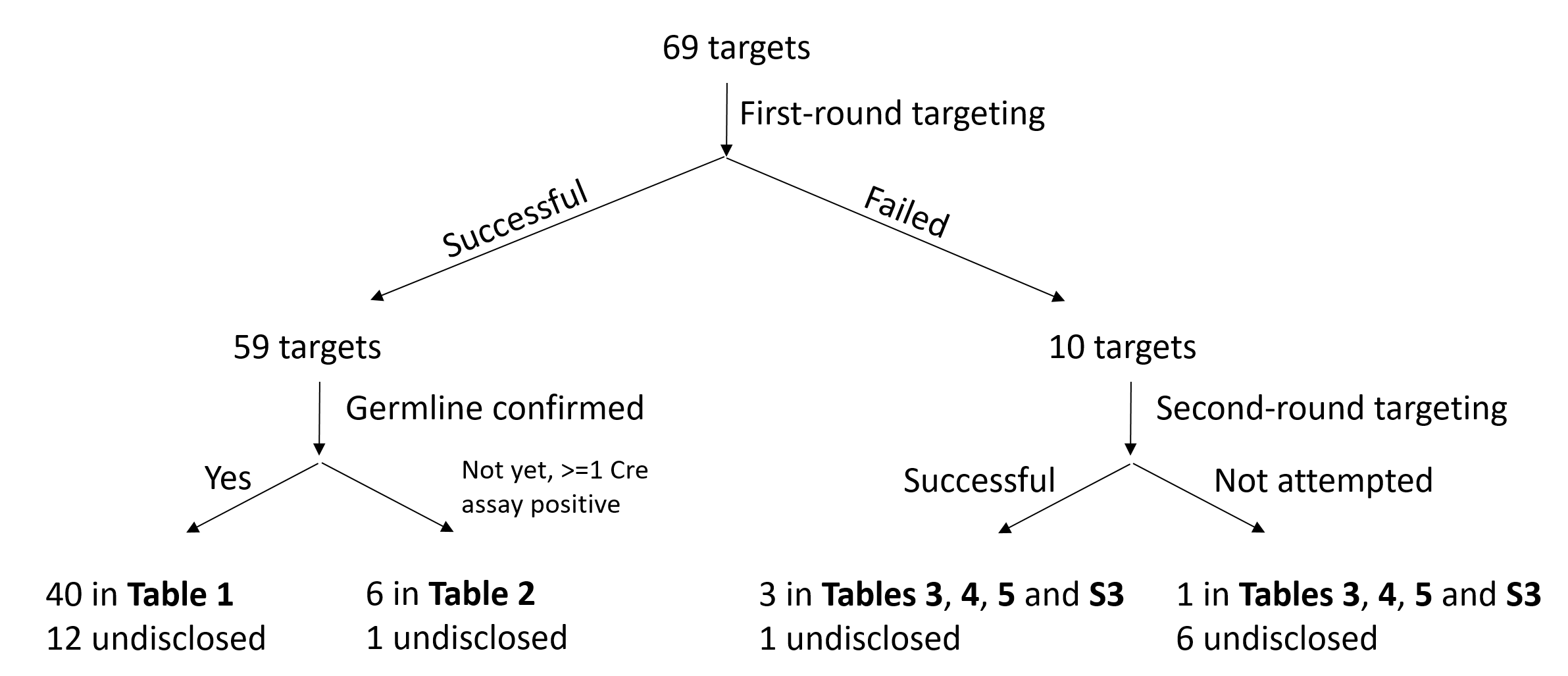


**Figure S1** Outcome of the 69 targets included in this study.


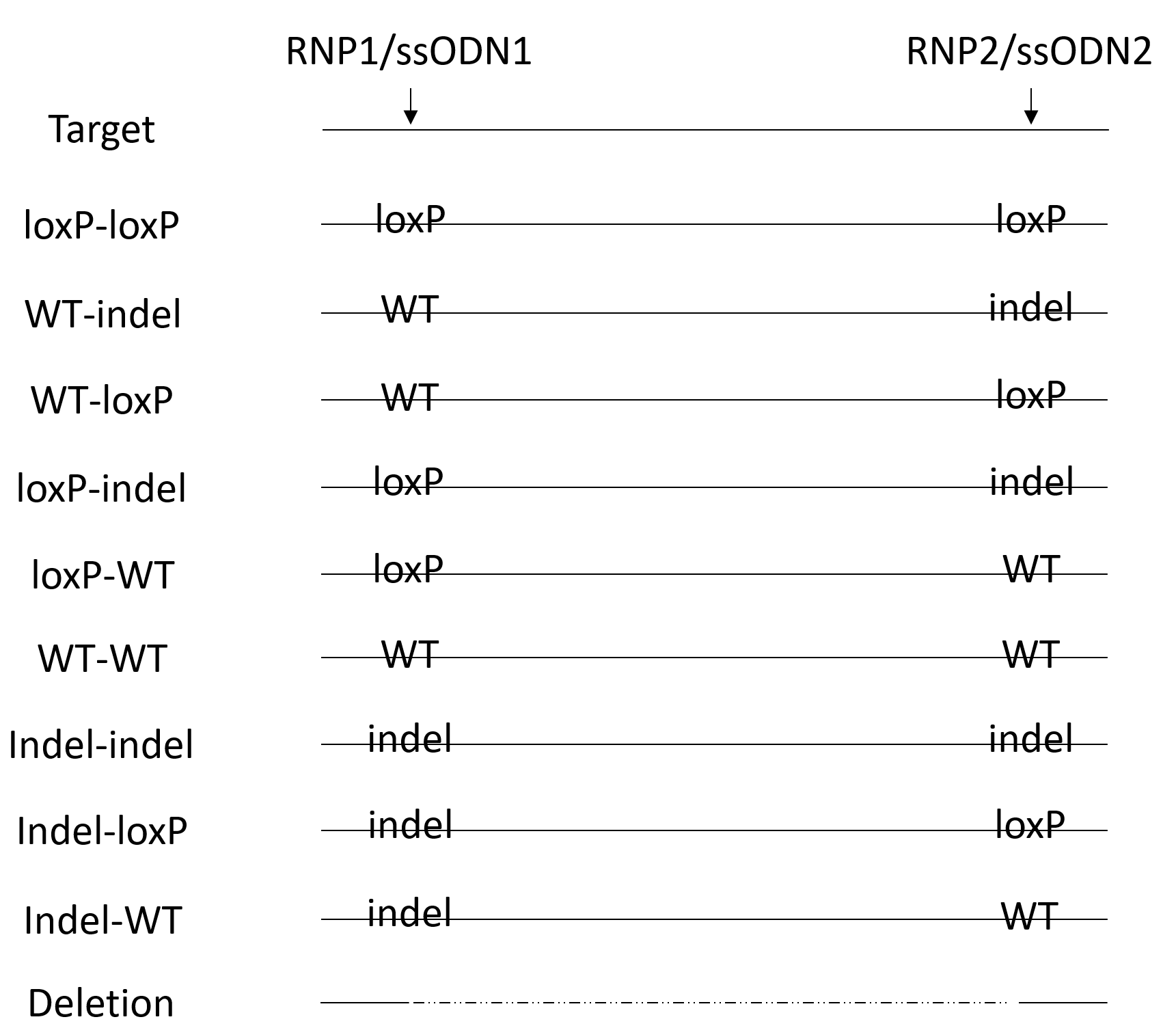


**Figure S2** Different possible editing outcomes after two RNPs and two ssODNs are introduced into the embryos.
